# Supplementary material for: A genome-scale metabolic model of Saccharomyces cerevisiae that integrates expression constraints and reaction thermodynamics
Source: Nat Commun. 2021 Aug 9;12:4790. doi: 10.1038/s41467-021-25158-6 (PMC8352978; doi:10.1038/s41467-021-25158-6)
Supplement: Supplementary file 1 — Supplementary Information [file 41467_2021_25158_MOESM1_ESM.pdf]

Supplementary Information

A genome-scale metabolic model of *Saccharomyces cerevisiae* that integrates expression constraints and reaction thermodynamics

Oftadeh *et al.*

Supplementary Notes

Implementing multiple expression systems

Since an important difference between bacterial and eukaryotic cells is the presence of multiple expression systems in the latter, we extended the original ETFL formulation to be able to consider multiple expression systems. Consider the following two constraints from the original formulation:

$$\sum_{l} P_l = E_{\text{RNAP}} \tag{1}$$

$$\sum_{l} R_l = E_{\text{rib}} \tag{2}$$

where R_l and P_l represent the amounts of ribosome and RNA polymerase that are allocated to transcribe and translate the gene l, respectively. Equation (1) implies that the sum of the RNA polymerases allocated to transcribe different peptides must be equal to total available RNA polymerases, i.e., E_{RNAP}. Similarly, Equation (2) implies that the sum of the ribosomes allocated to translate different peptides must be equal to total available ribosomes, i.e., E_{rib}. These constraints assume that all the genes are transcribed and translated by a single pool of RNA polymerase and ribosome, respectively. However, there are multiple types of RNA polymerase and ribosome in eukaryotes.

Without loss of generality and to make it clearer, we explain the extension for the case that there exist two types (pools) of RNA polymerase. Similar procedure can be applied to the cases that the number of RNA polymerases is different. Also, multiple types of ribosomes can be defined in the same way. Suppose that the cell has two RNA polymerases, RNAP_1 and RNAP_2. Per each type of RNA polymerase, an enzymatic pool can be considered, which is represented as $E^i_{\text{RNAP}}, i \in \{1,2\}$. The genes can be divided into at most three sets: (i) the genes transcribed by RNAP_1, represented by I_1, (ii) the genes transcribed by RNAP_2, represented by I_2 and (iii) the genes transcribed by both enzymes, represented by $I_{1,2}$. Instead of Equation (1), the following constraints are applied:

$$\sum_{l \in I_1} P_l \le E^1_{\text{RNAP}} \tag{3}$$

$$\sum_{l \in I_2} P_l \le E^2_{\text{RNAP}} \tag{4}$$

$$\sum_{l \in \{I_1 \cup I_2 \cup I_{1,2}\}} P_l = E_{\text{RNAP}}^1 + E_{\text{RNAP}}^2 \quad (5)$$

The Equation (3) and Equation (4) ensure that, respectively, the sum of the allocated RNA polymerase to the genes in the set I_1 and I_2 cannot exceed the total available RNA polymerase in the pool. The equality in Equation (1) is converted to inequality in these equations to let part of the RNA polymerases be allocated to the genes in the set $I_{1,2}$. Then, the Equation (5) enforces the equality between the total allocated RNA polymerases and the available enzyme in the two pools. By maximizing growth rate subject to these constraints, the cheaper RNA polymerase, i.e., the one with the less metabolic demand, takes over all the genes from the shared set ($I_{1,2}$).

Thermodynamic curation of the *S. cerevisiae* genome-scale model Yeast8

Including thermodynamic information in the *S. cerevisiae* GEM Yeast8 [1] allows us to constrain the reaction directionalities according to the second law of thermodynamics, that is, according to the Gibbs free energy of each reaction. Towards this end, we performed a thermodynamic curation of Yeast8. This curation was performed in two stages. In the first stage, we estimated the Gibbs free energy of formation of the compounds and the corresponding error for the estimation. First, we used MetaNetX (http://www.metanetx.org) [2] to annotate the compounds of Yeast8 with identifiers from public databases, such as KEGG [3], CHEBI [4], and SEED [5]. From these databases we obtained the structures of the compounds in the form of SMILES. For the compounds lacking SMILES, we manually derived them based on the structure and composition of the metabolites, including mainly lipids and phospholipids. We next used Marvin (version 18.1, 2018, ChemAxon http://www.chemaxon.com) to transform the SMILES into their major protonation state at pH 7 and to generate MDL Molfiles. These last ones were used in the Group Contribution Method (GCM) to estimate the standard Gibbs free energy of the formation of the compounds as well as the error of the estimation [6].

Following the described steps, we estimated the standard Gibbs free energy of formation for 1092 of 1326 total unique metabolites from Yeast8.

In the second stage, we estimated the Gibbs free energy of the reactions that are part of the Yeast8. First, we integrated in the Yeast8 model the thermodynamic properties of the compartments present in the model, including pH, ionic strength, membrane potentials and generic compartment concentration ranges (Table S5 and Table S6). Then, the standard Gibbs free energies of formation are adjusted following the Debye-Hükel approximation to the

corresponding pH and ionic strength [7]. The corrected Gibbs free energies of formation were then used to compute Gibbs free energy of reactions. Considering a reaction with m components, its Gibbs free energy, $\Delta_r \mathrm{G}'$ is:

$$\Delta_r \mathrm{G}' = \sum_{j=1}^{m} n_j \Delta_f \mathrm{G}_j'^o + RT \ln\left(\prod_{j=1}^{m} x_j^{n_j}\right), \tag{15}$$

where $j = 1, \dots, m$. n_j is the stoichiometric coefficient of compound j; $\Delta_f \mathrm{G}_j'^o$ is the standard Gibbs free energy of formation of compound j; x_j is the concentration of the compound j; R is the ideal gas constant, $R = 8.31 \cdot 10^{-3} \frac{KJ}{K\,mol}$, and T is the temperature. In this case, $T = 298\,K$.

In the case of transport reactions, the $\Delta_r \mathrm{G}'$ is corrected to account for the differences in pH and membrane potential between the compartments [7].

It is worth noting here, that these calculations and estimations of Gibbs free energies were performed for compounds and reactions under aqueous conditions. The Yeast8 model contains compounds that are part of membranes, and therefore they are no longer in an aqueous environment. We did not estimate $\Delta_r \mathrm{G}'$ for 1304 reactions that did not take place in aqueous environment, which resulted in a thermodynamic curation of 1880 out of 3991 reactions in the Yeast8 GEM.

In its current version, protons can freely circulate between the cytosol and the mitochondria in Yeast8. Seeking to have an improvement of the transport of protons across the mitochondrial membrane, we introduced in the Yeast8 GEM an additional compartment, to account for the mitochondrial inner-membrane space. This improvement allows to correctly capture the transport of protons in the electron transport chain (ETC) for the production of ATP by the enzyme ATP Synthase. Furthermore, this adjustment of proton transport will improve the estimation of the Gibbs free energy of the corresponding reactions as it will allow to account for the correct thermodynamic properties of the compartments involved (i.e., pH, ionic strength and membrane potential).

The thermodynamically curated Yeast8 model as well as the thermodynamic information (Gibbs free energy of formation for the compounds) are publicly available in Zenodo 10.5281/zenodo.4778047.

Comparing ETFL and the previous formulation of ME-models

In this section, we provide a general comparison between the ETFL [25] and the other ME-model formulations [23,24].

A key difference between the two formulations is that ETFL enables the integration of thermodynamic constraints and metabolomics data. Moreover, ETFL can account for multiple expression systems. On the other hand, the stable RNA splicing, and transcription initiation are only formulated in the other ME-model formulations. These functions can be also included in ETFL by expanding the formulation around the related processes. The transcription initiation is not completely neglected in ETFL but instead lumped in the other processes. This lumping is similar to the lumping used for translation initiation and elongation in all ME-models. Since it was shown that including such processes does not impact the predictions of the model in the protein limitation studies [24], and the transcription initiation is more complex in eukaryotic organisms, we did not model them at this stage. Also, in the other formulations of ME-models, the formation of metalloproteins is explicitly considered. In ETFL, the ionic requirements are lumped in the biomass reaction as it was done in the FBA models.

There were two formulations in the other ME-models regarding the enzyme mass balances. In the first formulation, presented in O'brien *et al.* [23], the enzymes appeared as metabolites in the reactions, and to properly account for mass balances, two pseudometabolites per enzyme were added to the model. Consider this toy example, where the following reaction with flux v_1 is catalyzed by enzyme E:

$$v_1: a \overset{E}{\rightarrow} b \quad (6)$$

To associate the enzyme abundance to the flux v_1, the following reactions were added:

$$v_0: \text{enzyme} \rightarrow \text{enzyme}_{\text{prime}} + \alpha\ \text{coupling} \quad (7)$$

$$v_1: a + \text{enzyme}_{\text{prime}} \rightarrow b + \text{enzyme} \quad (8)$$

$$v_2: \text{enzyme} + \text{coupling} \rightarrow \emptyset \quad (9)$$

where coupling and $\text{enzyme}_{\text{prime}}$ are pseudometabolites introduced to facilitate the addition of constraints and $\alpha = \frac{\mu}{k_{\text{cat}}}$. While the mass balance constraint associated with $\text{enzyme}_{\text{prime}}$ is an equality, the mass balance for coupling is an inequality. Writing the mass balances for the enzyme and the pseudometabolites, the following constraints can be derived:

$$v_{\text{tnsl}} - v_{\text{deg}} - v_2 = 0 \quad (10)$$

$$v_1 - v_0 = 0 \quad (11)$$

$$v_2 \geq \alpha v_0 \quad (12)$$

where v_{tnsl} and v_{deg} are respectively the rates of translation and degradation of the enzyme.

In the second formulation of ME-models, presented in Lloyd *et al.* [24], to simplify the solving process, the inequality constraint in Equation 12 was converted to an equality. This assumes that all the enzymes catalyze their reactions with the maximum possible rate at the optimal solution. This way, the two pseudometabolites and their corresponding reactions were removed.

In the ETFL formulation, no pseudometabolite is considered and the enzymes are not involved in the reactions as metabolites [25]. For the reaction presented by Equation 6, the following two constraints are imposed:

$$v_{\mathrm{tnsl}} - v_{\mathrm{deg}} - v_{\mathrm{dil}} = v_{\mathrm{tnsl}} - v_{\mathrm{deg}} - \mu E = 0 \qquad (13)$$

$$v_1 \leq k_{\mathrm{cat}} E \qquad (14)$$

where E is the total concentration of the enzyme and Equation 14 applies the generalized maximum flux (i.e., $V_{\mathrm{max}} = k_{\mathrm{cat}} E$). Although the constraints in Equations 13-14 differ from the ones in Equations 10-12 and the number of variables and constraints are different, it is straightforward to demonstrate that ETFL formulation and the formulation presented in O'brien *et al.* are mathematically equivalent. That is, from Equation 10 and Equation 13, it follows that $v_2 = \mu E$. Then, by replacing v_2 in Equation 12, we obtain Equation 14.

The equality assumption in Equation 12 presented in Lloyd *et al.* would be equivalently modeled in ETFL by converting the inequality in Equation 14 to an equality. This implies that at the optimal solution, all the enzymes perform under complete saturation, which might be an unrealistic assumption in some cases.

Supplementary Tables

Table S1: The scalar parameters used in yETFL

Parameter	Value and Unit	Reference
GC ratio	38.3 %	[8]
Length of the chromosome	12157105 bp	[9]
Median protein half-life	8.8 h	[10]
Median mRNA half-life	23 min	[11]
Median rRNA half-life	5 days	[12]
Average mRNA length	1250 nt	[13]
Median k_{cat}	70.9 s^{-1}	[14]
Average translation rate	10 (2-10) aa/s	[15]
Average transcription rate	40 nt/s	[16]
Fraction of proteome allocated to metabolism and expression	55 %	[17]
Ribosome occupancy ratio	85 %	[18]
RNA polymerase occupancy ratio	25 %	[19]

Table S2: The list of constraints in yETFL

Constraints		Description
**Expression Constraints**		
$\sum_j s_{ij}\,(v_j^f - v_j^b) = 0,$	$\forall i \in \mathrm{Met}$	Mass balances for the metabolites
$v_k^{\mathrm{asm}} - k_k^{\mathrm{deg}} E_k - \mu E_k = 0,$	$\forall k \in \mathrm{Enz}$	Mass balances for the enzymes
$v_l^{\mathrm{tnl}} - \sum_j \eta_j^l\, v_j^{\mathrm{asm}} = 0,$	$\forall l \in \mathrm{Pep}$	Mass balances for the peptides
$v_l^{\mathrm{tcp}} - k_l^{\mathrm{deg}} F_l - \mu F_l = 0,$	$\forall l \in \mathrm{mRNA}$	Mass balances for the mRNAs
$v_{aa_i}^{\mathrm{charging}} - \sum_l \eta_{aa_i}^l\, v_l^{tnl} - \mu T_{aa_i}^c = 0,$	$\forall aa_i \in \mathrm{AA}$	Mass balances for the charged tRNAs
$-v_{aa_i}^{\mathrm{charging}} + \sum_l \eta_{aa_i}^l\, v_l^{tnl} - \mu T_{aa_i}^u = 0,$	$\forall aa_i \in \mathrm{AA}$	Mass balances for the uncharged tRNAs
$v_{rRNA_i}^{\mathrm{tcp}} - k_{rRNA_i}^{\mathrm{deg}} F_{rRNA_i} - \mu F_{rRNA_i} - \eta_{\mathrm{Rib}}^{rRNA_i} v_{\mathrm{Rib}}^{\mathrm{asm}} = 0,$	$\forall \mathrm{rRNA}_i \in \mathrm{rRNA}$	Mass balances for the rRNAs
$v_j^f \leq \sum_{k \in Enz_j} k_{\mathrm{cat},k}^f E_k,$	$\forall j \in \mathrm{Rxn}$	Forward catalytic efficiency
$v_j^b \leq \sum_{k \in Enz_j} k_{\mathrm{cat},k}^b E_k,$	$\forall j \in \mathrm{Rxn}$	Backward catalytic efficiency
$v_l^{tnl} \leq \frac{k_{\mathrm{cat}}^{\mathrm{Rib}}}{L_l^{aa}} R_l$	$\forall l \in \mathrm{Pep}$	Translation catalytic efficiency
$v_l^{tcp} \leq \frac{k_{\mathrm{cat}}^{\mathrm{RNAP}}}{L_l^{nt}} P_l$	$\forall l \in \mathrm{mRNA}$	Transcription catalytic efficiency
$R_l \leq \frac{L_l^{nt}}{L_{\mathrm{Rib}}} F_l$	$\forall l \in \mathrm{Pep}$	Translation coupling
$P_l \leq \frac{L_l^{nt}}{L_{\mathrm{RNAP}}} n_l \mathrm{DNA}$	$\forall l \in \mathrm{mRNA}$	Transcription coupling
$\sum_l P_l = E_{\mathrm{RNAP}}$		RNA polymerase total capacity

$\sum_{l \in I_A} R_l \leq E_{\text{Rib}}^A$		Ribosome A capacity
$\sum_{l \in I_B} R_l \leq E_{\text{Rib}}^B$		Ribosome B capacity
$\sum_{l \in \{I_A \cup I_B \cup I_{A,B}\}} R_l = E_{\text{Rib}}^A + E_{\text{Rib}}^B$		Ribosome total capacity
$\sum_{j \neq \text{dummy protein}} \text{MW}_j E_j = \varphi \cdot P^m$		ME-enzyme allocation
**Thermodynamic Constraints**		
$-\Delta_r G_j' + RT \sum_{i=1}^{m} s_{ij} C_i + \Delta_r G_j'^{\circ} = 0$	$\forall j \in \text{Rxn}_G$	Calculation of the free Gibbs energy
$\Delta_r G_j' \leq M(1 - b_j^f)$	$\forall j \in \text{Rxn}_G$	Forward Gibbs energy coupling
$-\Delta_r G_j' \leq M(1 - b_j^b)$	$\forall j \in \text{Rxn}_G$	Backward Gibbs energy coupling
$0 \leq v_j^f \leq M b_j^f$	$\forall j \in \text{Rxn}$	Forward directionality coupling
$0 \leq v_j^b \leq M b_j^b$	$\forall j \in \text{Rxn}$	Backward directionality coupling
$b_j^f + b_j^b \leq 1$	$\forall j \in \text{Rxn}$	Simultaneous use of directionalities
**Constant Biomass Constraints**		
$\sum_j \text{MW}_j E_j = P^m$		Total protein allocation
$\sum_l \text{MW}_l F_l = R^m$		Total RNA allocation
**Variable Biomass Constraints**		
$\sum_j \text{MW}_j E_j = \sum_u \lambda_u P_u^m$		Total protein allocation
$\sum_l \text{MW}_l F_l = \sum_u \lambda_u R_u^m$		Total RNA allocation

$\mathrm{MW_{DNA}DNA} = \sum_{u} \lambda_u D_u^m$		DNA allocation
$\eta_{\mathrm{bbb}_i,\ ref}^{\mathrm{biomass}} \frac{X_{u,\mathrm{bbb}_i}^m}{X_{\mathrm{ref,\,bbb}_i}^m} \mu - \eta_{\mathrm{bbb}_i}^{\mathrm{pool}} v_{\mathrm{bbb}_i}^{\mathrm{poo}l} = 0,$	$\forall \mathrm{bbb}_i \in \mathrm{BBB}$	Mass balances for the other biomass building blocks

Table S3: definition of the variables, parameters and sets used in Table S2.

Symbol	Definition
μ	Specific growth rate
v_j^f	Flux of the reaction j in forward direction
v_j^b	Flux of the reaction j in backward direction
v_k^{asm}	Flux of the assembly of the enzyme complex k
v_l^{tcp}	Flux of the transcription of the mRNA l
v_l^{tnl}	Flux of the translation of the peptide l
$v_{aa_i}^{\text{charging}}$	Flux of charging the tRNAs with the amino acid aa_i
F_l	Concentration of mRNA l
E_k	Concentration of enzyme k
DNA	Concentration of DNA
P_l	RNA polymerase concentration allocated to transcription of the mRNA l
R_l	Ribosome concentration allocated to translation of the peptide l
$T_{aa_i}^c$	Concentration of tRNAs charged with amino acid aa_i
$T_{aa_i}^u$	Concentration of tRNAs uncharged with amino acid aa_i
C_i	Concentration of metabolite i
$\Delta_r G_j'$	Free Gibbs energy of the reaction j
$\Delta_r G_j'^{\circ}$	Standard free Gibbs energy of the reaction j
b_j^f	Binary variable to represent if the forward direction is active for reaction j

b_j^b	Binary variable to represent if the backward direction is active for reaction j
λ_u	Binary variables to determine which growth bin is active
$k_{\mathrm{cat},k}^f$	Turnover number of the enzyme k in the forward direction
$k_{\mathrm{cat},k}^b$	Turnover number of the enzyme k in the backward direction
k_*^{deg}	Degradation constant of the macromolecule *
$k_{\mathrm{cat}}^{\mathrm{Rib}}$	Ribosomal elongation rate
$k_{\mathrm{cat}}^{\mathrm{RNAP}}$	RNA polymerase elongation rate
P^m	Fraction of the cell that is protein
R^m	Fraction of the cell that is RNA
D^m	Fraction of the cell that is DNA
φ	Fraction of the proteome that is metabolism and expression enzymes
MW_*	Molecular weight of the macromolecule *
R	Universal gas constant
T	Temperature
M	An arbitrarily large number
L_l^{nt}	Length of the mRNA l
L_l^{aa}	Length of the peptide l
L_{Rib}	Ribosomal footprint
L_{RNAP}	RNA polymerase footprint
Met	Set of all metabolites
Rxn	Set of all reactions
Rxn_G	Set of reactions with defined Gibbs energy
Enz	Set of all enzymes

Pep	Set of all peptides
mRNA	Set of all mRNAs
rRNA	Set of all rRNAs
AA	Set of all amino acids
BBB	Set of all biomass building blocks

Table S4: Different amino acid ratios in *Saccharomyces cerevisiae* [20]

**Amino acid**	**Fraction (%)**
Ala	9.77
Arg	3.86
Asx	9.28
Cys	0.14
Glx	15.48
Gly	8.89
His	1.93
Ile	5.89
Leu	8.01
Lys	6.57
Ala	9.77
Arg	3.86
Met	1.14
Phe	3.76
Pro	4.22
Ser	5.33
Thr	5.57
Trp	0.65
Tyr	1.96
Val	7.33

Table S5: Biomass composition of *Saccharomyces cerevisiae* at different growth rates [20-22]

mu	Protein	RNA	Carbohydrate	Lipid	DNA	Ion	Water	Pi
0.022	34.5	3.8	44.9	8	0.4	3	4.1	1.4
0.052	38.5	4.6	40.9	7.8	0.4	2.7	3.9	1.2
0.087	39.7	5.4	40.6	6.2	0.4	3	3.7	1.1
0.107	40.7	6.6	38.7	6.1	0.3	2.7	4.1	0.8
0.126	40.1	5.7	38.8	7	0.4	2.7	4.4	1
0.158	42.2	5.9	35.9	7	0.4	2.9	4.8	0.9
0.211	43.8	6.6	34.4	6.7	0.4	2.9	4.4	0.7
0.3	48	9.4	30.1	7	0.4	2.9	1.5	0.7
0.35	51.9	10.2	27.7	7	0.4	2.9	0	0
0.4	52.4	11	25.4	7	0.4	2.9	0.2	0.7

Table S6: Corrected physicochemical specification of different compartments in Yeast8

Compartment Name	Compartment Symbol	pH	Ionic Strength	Concentration Range (M)
cytoplasm	c	7.0000	0.25	10^{-6} - $5\text{x}10^{-2}$
mitochondrion	m	7.5000	0.25	10^{-6} - $5\text{x}10^{-2}$
mitochondrial membrane	mm	7.5000	0.25	10^{-6} - $5\text{x}10^{-2}$
vacuole	v	6.1700	0	10^{-6} - $5\text{x}10^{-2}$
vacuolar membrane	vm	6.1700	0	10^{-6} - $5\text{x}10^{-2}$
peroxisome	p	8.2000	0	10^{-6} - $5\text{x}10^{-2}$
extracellular	e	5.0000	0	10^{-8} - 10^{-1}
Golgi	g	6.3500	0	10^{-6} - $5\text{x}10^{-2}$
Golgi membrane	gm	6.3500	0	10^{-6} - $5\text{x}10^{-2}$
endoplasmic reticulum	er	7.2000	0	10^{-6} - $5\text{x}10^{-2}$
endoplasmic reticulum membrane	erm	7.2000	0	10^{-6} - $5\text{x}10^{-2}$
nucleus	n	7.0000	0	10^{-6} - $5\text{x}10^{-2}$
lipid particle	lp	7.0000	0.25	10^{-6} - $5\text{x}10^{-2}$
cell envelope	ce	7.0000	0	10^{-6} - $5\text{x}10^{-2}$
inter-membrane mitochondrion	im	7.0000	0	10^{-6} - $5\text{x}10^{-2}$

Table S7: membrane potentials for different compartments in Yeast8 (compartment symbols are used from Table S5)

	c	m	mm	v	vm	p	e	g	gm	er	erm	n	lp	ce	im
c	0	-180	-180	0	0	0	60	0	0	0	0	-15	0	0	0
m	180	0	0	0	0	0	0	0	0	0	0	0	0	0	0
mm	180	0	0	0	0	0	0	0	0	0	0	0	0	0	0
v	0	0	0	0	0	0	0	0	0	0	0	0	0	0	0
vm	0	0	0	0	0	0	0	0	0	0	0	0	0	0	0
p	0	0	0	0	0	0	0	0	0	0	0	0	0	0	0
e	-60	0	0	0	0	0	0	0	0	0	0	0	0	0	0
g	0	0	0	0	0	0	0	0	0	0	0	0	0	0	0
gm	0	0	0	0	0	0	0	0	0	0	0	0	0	0	0
er	0	0	0	0	0	0	0	0	0	0	0	0	0	0	0
erm	0	0	0	0	0	0	0	0	0	0	0	0	0	0	0
n	15	0	0	0	0	0	0	0	0	0	0	0	0	0	0
lp	0	0	0	0	0	0	0	0	0	0	0	0	0	0	0
ce	0	0	0	0	0	0	0	0	0	0	0	0	0	0	0
im	0	0	0	0	0	0	0	0	0	0	0	0	0	0	0

Supplementary References

1. Lu, H., et al., *A consensus S. cerevisiae metabolic model Yeast8 and its ecosystem for comprehensively probing cellular metabolism.* Nature communications, 2019. **10**(1): p. 1-13.
2. Moretti, S., et al., *MetaNetX/MNXref–reconciliation of metabolites and biochemical reactions to bring together genome-scale metabolic networks.* Nucleic acids research, 2016. **44**(D1): p. D523-D526.
3. Kanehisa, M. and S. Goto, *KEGG: kyoto encyclopedia of genes and genomes.* Nucleic acids research, 2000. **28**(1): p. 27-30.
4. Degtyarenko, K., et al., *ChEBI: a database and ontology for chemical entities of biological interest.* Nucleic acids research, 2007. **36**(suppl_1): p. D344-D350.
5. Devoid, S., et al., *Automated genome annotation and metabolic model reconstruction in the SEED and Model SEED*, in *Systems Metabolic Engineering*. 2013, Springer. p. 17-45.
6. Mavrovouniotis, M.L., *Group contributions for estimating standard Gibbs energies of formation of biochemical compounds in aqueous solution.* Biotechnology and bioengineering, 1990. **36**(10): p. 1070-1082.
7. Weilandt, D.R., M. Masid, and V. Hatzimanikatis, *Thermodynamics of Metabolic Pathways*. 2021: Metabolic Engineering: Concepts and Applications.
8. Wood, V., et al., *The genome sequence of Schizosaccharomyces pombe.* nature, 2002. **415**(6874): p. 871-880.
9. Goffeau, A., et al., *Life with 6000 genes.* Science, 1996. **274**(5287): p. 546-567.
10. Christiano, R., et al., *Global proteome turnover analyses of the yeasts S. cerevisiae and S. pombe.* Cell reports, 2014. **9**(5): p. 1959-1965.
11. Wang, Y., et al., *Precision and functional specificity in mRNA decay.* Proceedings of the National Academy of Sciences, 2002. **99**(9): p. 5860-5865.
12. Planta, R.J. and W.H. Mager, *The list of cytoplasmic ribosomal proteins of Saccharomyces cerevisiae.* Yeast, 1998. **14**(5): p. 471-477.
13. Miura, F., et al., *Absolute quantification of the budding yeast transcriptome by means of competitive PCR between genomic and complementary DNAs.* BMC genomics, 2008. **9**(1): p. 1-14.
14. Sánchez, B.J., et al., *Improving the phenotype predictions of a yeast genome-scale metabolic model by incorporating enzymatic constraints.* Molecular systems biology, 2017. **13**(8): p. 935.
15. Karpinets, T.V., et al., *RNA: protein ratio of the unicellular organism as a characteristic of phosphorous and nitrogen stoichiometry and of the cellular requirement of ribosomes for protein synthesis.* BMC biology, 2006. **4**(1): p. 30.
16. Koš, M. and D. Tollervey, *Yeast pre-rRNA processing and modification occur cotranscriptionally.* Molecular cell, 2010. **37**(6): p. 809-820.
17. Wang, M., et al., *Version 4.0 of PaxDb: protein abundance data, integrated across model organisms, tissues, and cell-lines.* Proteomics, 2015. **15**(18): p. 3163-3168.
18. Zenklusen, D., D.R. Larson, and R.H. Singer, *Single-RNA counting reveals alternative modes of gene expression in yeast.* Nature structural & molecular biology, 2008. **15**(12): p. 1263.
19. Ehrenberg, M., H. Bremer, and P.P. Dennis, *Medium-dependent control of the bacterial growth rate.* Biochimie, 2013. **95**(4): p. 643-658.

20. Lange, H. and J. Heijnen, *Statistical reconciliation of the elemental and molecular biomass composition of Saccharomyces cerevisiae.* Biotechnology and bioengineering, 2001. **75**(3): p. 334-344.
21. Gombert, A.K., et al., *Network identification and flux quantification in the central metabolism of Saccharomyces cerevisiae under different conditions of glucose repression.* Journal of bacteriology, 2001. **183**(4): p. 1441-1451.
22. Van Hoek, P., J.P. Van Dijken, and J.T. Pronk, *Effect of specific growth rate on fermentative capacity of baker's yeast.* Appl. Environ. Microbiol., 1998. **64**(11): p. 4226-4233.
23. O'brien, E.J., Lerman, J.A., Chang, R.L., Hyduke, D.R. & Palsson, B.Ø. Genome-scale models of metabolism and gene expression extend and refine growth phenotype prediction. *Molecular systems biology* **9** (2013).
24. Lloyd, C.J. et al. COBRAme: A computational framework for genome-scale models of metabolism and gene expression. *Plos Comput Biol* **14**, e1006302 (2018).
25. Salvy, P. & Hatzimanikatis, V. The ETFL formulation allows multi-omics integration in thermodynamics-compliant metabolism and expression models. *Nat Commun* **11**, 1-17 (2020).
