# Supplementary material for: A genome-scale metabolic model of Saccharomyces cerevisiae that integrates expression constraints and reaction thermodynamics
Source: Nat Commun. 2021 Aug 9;12:4790. doi: 10.1038/s41467-021-25158-6 (PMC8352978; doi:10.1038/s41467-021-25158-6)
Supplement: Supplementary file 3 — Reporting Summary [file 41467_2021_25158_MOESM3_ESM.pdf]

nature research

Corresponding author(s): Vassily Hatzimanikatis

Last updated by author(s): Jul 11, 2021

Reporting Summary

Nature Research wishes to improve the reproducibility of the work that we publish. This form provides structure for consistency and transparency in reporting. For further information on Nature Research policies, see our Editorial Policies and the Editorial Policy Checklist.

Statistics

For all statistical analyses, confirm that the following items are present in the figure legend, table legend, main text, or Methods section.

n/a	Confirmed	
☒	☐	The exact sample size (*n*) for each experimental group/condition, given as a discrete number and unit of measurement
☒	☐	A statement on whether measurements were taken from distinct samples or whether the same sample was measured repeatedly
☒	☐	The statistical test(s) used AND whether they are one- or two-sided<br>*Only common tests should be described solely by name; describe more complex techniques in the Methods section.*
☒	☐	A description of all covariates tested
☒	☐	A description of any assumptions or corrections, such as tests of normality and adjustment for multiple comparisons
☒	☐	A full description of the statistical parameters including central tendency (e.g. means) or other basic estimates (e.g. regression coefficient) AND variation (e.g. standard deviation) or associated estimates of uncertainty (e.g. confidence intervals)
☒	☐	For null hypothesis testing, the test statistic (e.g. F, t, r) with confidence intervals, effect sizes, degrees of freedom and P value noted<br>*Give P values as exact values whenever suitable.*
☒	☐	For Bayesian analysis, information on the choice of priors and Markov chain Monte Carlo settings
☒	☐	For hierarchical and complex designs, identification of the appropriate level for tests and full reporting of outcomes
☒	☐	Estimates of effect sizes (e.g. Cohen's *d*, Pearson's *r*), indicating how they were calculated

Our web collection on statistics for biologists contains articles on many of the points above.

Software and code

Policy information about availability of computer code

Data collection	Custom code has been written in Python 3.7, and the commercial solver Gurobi was used to solve the MILP problems. The code to generate yETFL models and reproduce the results of this paper is freely available at https://github.com/EPFL-LCSB/yetfl and https://gitlab.com/EPFL-LCSB/yetfl. The code relies on the ETFL (https://github.com/EPFL-LCSB/etfl) and pyTFA (https://github.com/EPFL-LCSB/pytfa) packages, which use COBRApy and Optlang.
Data analysis	Data analysis has been done with Python 3.7 and the scripts to generate the results are available on our GitHub and GitLab repository

For manuscripts utilizing custom algorithms or software that are central to the research but not yet described in published literature, software must be made available to editors and reviewers. We strongly encourage code deposition in a community repository (e.g. GitHub). See the Nature Research guidelines for submitting code & software for further information.

Data

Policy information about availability of data

All manuscripts must include a data availability statement. This statement should provide the following information, where applicable:
- Accession codes, unique identifiers, or web links for publicly available datasets
- A list of figures that have associated raw data
- A description of any restrictions on data availability

The supporting data is available in https://doi.org/10.5281/zenodo.4778047. The data has been obtained from public databases including KEGG (https://www.genome.jp/kegg), yeastCyc (https://yeast.biocyc.org), and Complex Portal (https://www.ebi.ac.uk/complexportal/home) and publications, for which the references are provided in the main text.

Field-specific reporting

Please select the one below that is the best fit for your research. If you are not sure, read the appropriate sections before making your selection.

[x] Life sciences   [ ] Behavioural & social sciences   [ ] Ecological, evolutionary & environmental sciences

For a reference copy of the document with all sections, see nature.com/documents/nr-reporting-summary-flat.pdf

Life sciences study design

All studies must disclose on these points even when the disclosure is negative.

Sample size	No samples were taken in this study.
Data exclusions	No data was excluded from the study.
Replication	MILP optimal values are a global optimum, and are thus mathematically guaranteed to be replicated.
Randomization	There is no step in the formulation where randomization can be used.
Blinding	There is no step in the formulation where blinding can be used.

Reporting for specific materials, systems and methods

We require information from authors about some types of materials, experimental systems and methods used in many studies. Here, indicate whether each material, system or method listed is relevant to your study. If you are not sure if a list item applies to your research, read the appropriate section before selecting a response.

Materials & experimental systems

n/a	Involved in the study	
[x]	[ ]	Antibodies
[x]	[ ]	Eukaryotic cell lines
[x]	[ ]	Palaeontology and archaeology
[x]	[ ]	Animals and other organisms
[x]	[ ]	Human research participants
[x]	[ ]	Clinical data
[x]	[ ]	Dual use research of concern

Methods

n/a	Involved in the study	
[x]	[ ]	ChIP-seq
[x]	[ ]	Flow cytometry
[x]	[ ]	MRI-based neuroimaging
